# Improving the Behavioral Intention of Continuous Online Learning Among Learners in Higher Education During COVID-19

**DOI:** 10.3389/fpsyg.2022.857709

**Published:** 2022-04-26

**Authors:** Wei Xu, Zhi-Yi Shen, Shi-Jia Lin, Jia-Chen Chen

**Affiliations:** ^1^College of Educational Science and Technology, Zhejiang University of Technology, Hangzhou, China; ^2^College of Management, Zhejiang University of Technology, Hangzhou, China

**Keywords:** COVID-19, UTAUT2, online education, capability of metacognition and self-regulation, self-efficacy

## Abstract

The COVID-19 pandemic caused colleges and universities to rely heavily on online learning to continue knowledge dissemination to learners. This study used the second-generation model of unified theory of acceptance and use of technology (UTAUT2) to comprehensively analyze the mediating effects of *self-efficacy*, which affects learners’ effective use of online tools for learning, and *capability of metacognition and self-regulation*, which can independently adjust learning progress into the UTAUT2 model, on the learner’s willingness to continue online learning [i.e., their behavioral intention (BI)] by constructing a UTAUT2-based e-learning model. This study administered questionnaires to undergraduates in universities in East China to collect data. The effects of performance expectancy, effort expectancy (EE), social influence (SI), and facilitating conditions (FCs), hedonic motivation (HM), price value (PV), and habits on BI (directly or through mediators) were analyzed through data analysis and structural equation modeling, and the UTAUT2-based e-learning model was accordingly modified. The results indicated that the self-efficacy enhanced the effects of EE, SI, FCs, HM, and PV on learners’ BI; that metacognition and self-regulation (MS) capabilities enhanced the effects of EE on learners’ BI; and that habits had a direct and strong effect on BI. This study also provided some suggestions to enhance higher education learners’ willingness to continue online learning, such as improving social recognition and support, careful design of teaching content, easy-to-use technology, financial support. These results and suggestions may guide colleges and universities in conducting, continuing, or enhancing online education, particularly as the pandemic continues.

## Introduction

During the COVID-19 pandemic that commenced in 2020, online learning helped overcome the social distancing restrictions to ensure that educational activities at all levels and types of schools could proceed. Colleges and universities have been required to exploit the advantages of online learning and promote the integration of online and offline teaching (Zhong and Nan, 2021). The strict supervision of education departments can partly guarantee the quality of the course content and teaching implementation. Nevertheless, many challenges remain, such as low satisfaction with online learning, low participation, low readiness for online learning, low attention input, and poor completion rates (Li Y.Y. et al., 2020; Li Y. et al., 2020; Wan et al., 2020; Li et al., 2021). Some scholars have argued that external and objective aspects, such as teachers’ learning ability and teacher support, should be improved to enhance the learning outcomes of online teaching (Jia et al., 2021; Jing et al., 2021). However, irrespective of the optimization of external objective conditions, learners’ willingness to accept online learning and their internal psychological willingness to continue online learning constitute the core internal drive that determines learning outcomes (Clay et al., 2008).

The unified theory of acceptance and use of technology (UTAUT) can be used to predict the learner’s behavioral intention (BI) (Liu et al., 2007). The second-generation model of UTAUT (UTAUT2) has the highest explanatory power for the acceptance and continuous use of individual technology among the existing models (Venkatesh et al., 2012). Mittal et al. (2021) extended the UTAUT which can highlight the determinants of the adoption of online teaching during COVID-19 with three new constructs, namely facilitative leadership, regulatory support and project team capability. This study extended the UTAUT2 by incorporating self-efficacy (SE), which affects learners’ effective use of online tools for learning, and the capability of metacognition and self-regulation (MS), which can independently influence learning progress, as mediating variables. This extended model is called the UTAUT2-based e-learning model.

Tandon et al. (2021) identified the facilitators and inhibitors for the adoption of e-learning for the undergraduate students by structural equation modeling (SEM), found that all the identified facilitators emerged significant except social influence (SI) and price value (PV), and technology risk emerged insignificant while all other inhibitors had significant impact on BI to adopt e-learning. Several researchers from different countries have explore the effectiveness and learners’ perceptions of online learning in higher education during COVID-19 (Al-Karaki et al., 2021; Packmohr and Brink, 2021; Peimani and Kamalipour, 2021; Ranjan et al., 2021). The purpose of this study is to validate the UTAUT2-based e-learning model to comprehensively explore learners’ intention to continue online learning during COVID-19 in China, which is different from the previous studies. This study used questionnaires to collect data and a structural equation model (SEM) to verify the results and make suggestions for improving the learners’ willingness to continue online learning.

## Overview of UTAUT2

### Development From Unified Theory of Acceptance and Use of Technology to UTAUT2

**FIGURE 1:**
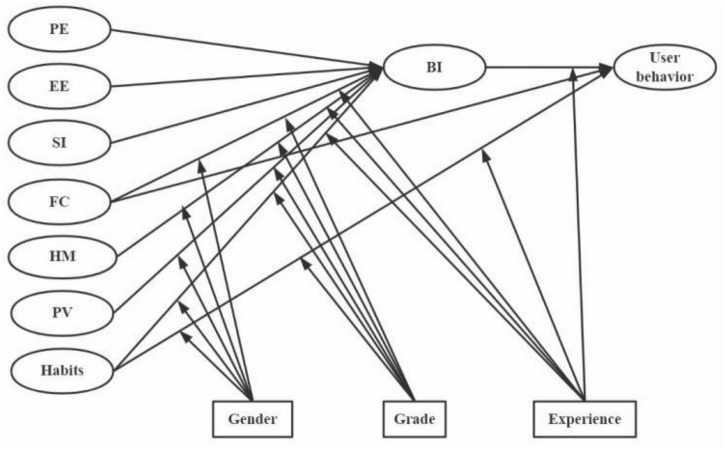
The UTAUT2 model.

Venkatesh et al. (2003) comprehensively analyzed eight models and extracted four independent variables—performance expectancy (PE), effort expectancy (EE), SI, and facilitating conditions (FCs)—to predict the BI and user behavior of users’ continuous use of certain technologies, thereby forming the UTAUT. Studies have explored various fields of technological application using diverse approaches, such as single model, model expansion, model combination, and model integration (Zhang et al., 2018). A meta-analysis of 161 studies published from 2007 to 2016, revealed that the four independent variables of the UTAUT had moderate or even low effect values on BI and user behavior (Han, 2017). Venkatesh et al. (2012), who first proposed the UTAUT, added three independent variables to construct the UTAUT2 model: hedonic motivation (HM), PV, and habits (Figure 1). Compared with the UTAUT, the explanatory power of BI increased from 56 to 74% and user behavior increased from 40 to 52% in UTAUT2, which is the highest among the existing models (Wong et al., 2013).

### Related Studies on Unified Theory of Acceptance and Use of Technology and UTAUT2

The UTAUT can predict BI in ordinary individuals and in learners (Liu et al., 2007; Yang et al., 2014). Table 1 provides a summary of related studies that employed the UTAUT model to predict BI of continued use for a technology or product.

**TABLE 1:** Related studies.

References	Technology or product	Influencing factor
Wong et al., 2013	Interactive electronic whiteboard	PE, EE, SI, FC, and applicability of the UTAUT model
Yang et al., 2014	E-learning	PE, EE, SI, FC, and information technology adoption
Wang and Mao, 2016	MOOC	PE, EE, SI, and FC
Yang, 2016	MOOC	Intrinsic motivation, basic psychological demand factors (perceptual autonomy, perceptual ability, perceptual relationship), MOOC design factors (content quality, autonomy, social interaction), extrinsic motivation (perceptual usefulness), satisfaction, and expectation confirmation
Shiau and Chau, 2016	Cloud-computing classroom	Attitude, compatibility, SE, quality of cloud service, perceived behavioral control, perceived ease of use, perceived interest, perceived usefulness, result presentation, subjective specification, quality of applied service, and visibility
Raja Yusof et al., 2017	Real-time visualization system using radio-frequency identification	PE, EE, SI, and FC
Chen and Hwang, 2019	Online learning behavior	Metacognition and self-regulation, motivational self-regulation, PE, EE, and SI
He and Zhang, 2019	College students’ English mobile learning	PE, EE, SI, and FC
Zeng, 2019	Mobile language learning	PE, EE, SI, FC, HM, PV, habits, experience, year of study, and major

Most studies have explored learners’ BI for using a technology or product based on the UTAUT model, but few have used the UTAUT2 model. Furthermore, multiple studies have integrated other influencing factors associated with learners, such as SE, metacognition, and self-regulation. Online learning relies on various synchronous and asynchronous teaching technology tools to achieve time and space flexibility. Teachers must provide a learning space for independent learning and meet the needs of learners for personalized learning. Therefore, the effect of online learning is largely dependent on the learners’ SE related to using technology tools and their ability for self-regulation.

## Research Design

### Model Reconstruction

Rodgers et al. (2002) verified the beneficial effect of SE on BI by using confirmatory factor analysis. Shiau and Chau (2016) employed a multimodel comparison approach and revealed that SE positively affected the BI of learners using cloud-computing classrooms. In addition, students have high SE and MS capabilities when learning online (Yuan, 2014). Chen and Hwang (2019) demonstrated that college students’ willingness to use networks was indirectly influenced by MS capabilities.

**FIGURE 2:**
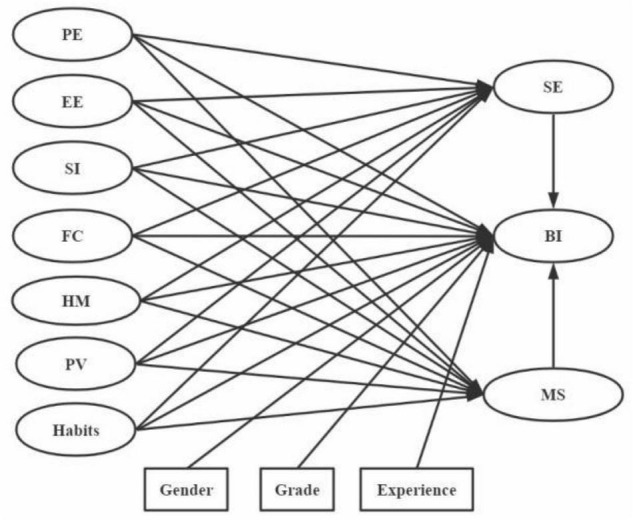
UTAUT2-based e-learning model.

Therefore, this study selected the UTAUT2 model, which has high explanatory power, and included SE and MS as the two mediating variables to explore their mediating effect on BI—that is, willingness of college learners to continue online learning. Considering that user behavior in the UTAUT2 model can be reflected by BI (Yang, 2016), this study considered BI as the independent variable. Figure 2 presents the reconstructed UTAUT2-based e-learning model.

In the UTAUT2-based e-learning model, the independent variables affecting the willingness of college learners to continue online learning were PE, EE, SI, FC, HM, PV, and habits; the mediating variables were SE and MS; and the relevant variables were gender, year of study, and experience.

Behavioral intention in this study was used to refer to the learners’ willingness to continue online learning both during subsequent routine learning or emergency situations when they must remain at home after online learning during the pandemic period.

Performance expectancy refers to an individual’s expectation that using a given technology would help improve their performance. In this study, PE was used to refer to the expectation of learners that online learning would improve their academic performance. The more the online learning meets their expectations, the stronger their BI is.

Effort expectancy refers to the difficulty of mastering a technology. In this study, EE was used to refer to the difficulty learners experience in mastering the use of online learning. When learners feel that technical problems in online learning can be addressed in a simple and rapid manner, BI is enhanced.

Social influence reflects the impact of others’ beliefs or the external environment on an individual’s willingness or behavior. In this study, SI was used to refer to the view of the learner’s social group regarding the learner’s online learning; if external factors are positive regarding the learner’s choice to learn online, BI is enhanced.

Facilitating condition refers to the degree of support that individuals believe they receive in terms of organization and technological systems for their use of new technologies. In this study, FC was used to refer to the ability of learners to obtain guidance on objective conditions, such as resources, technologies, and tools, used to support their online learning. The stronger the perceived guidance and support is, the higher the BI is.

Hedonic motivation refers to the individual using technology that stimulates their intrinsic motivation to improve BI. In this study, HM refers to the extent to which learners experience enjoyment during online learning. The more enjoyment they experience while learning, the stronger their BI is.

Price value refers to the trade-off between benefits and sacrifices. The stronger the BI is, the lower the monetary cost is for online learning and the greater the learning gain is.

Habits reflect the degree of automation of individual behavior. In this study, learners’ repeated engagement in online learning behavior or the cultivation of habits related to online learning has a positive impact on BI.

Self-efficacy refers to the degree of confidence that individuals have in their skills to complete a certain task, which is a non-intellectual factor. In this study, the higher the SE is, the stronger learners’ interest and motivation in online learning is for achieving learning goals, thus leading to higher BI.

MS refers to individuals’ subjective judgment of their cognitive activity and ability to acquire knowledge and skills and clarify their learning paths during online learning to improve their BI.

### Hypotheses

On the basis of the existing UTAUT2 model and related studies, this study proposed the following hypotheses:

H1: The UTAUT2-based e-learning model would explain the learners’ willingness to continue their online learning, and the factors would have significant effects on the dependent variable (BI).

H2: The effects of the independent variables on the dependent variable (BI) would be enhanced through the mediating effect of SE.

H3: The effects of the independent variables on the dependent variable (BI) would be enhanced through the mediating effect of MS.

### Tools

The questionnaire (49 items) was divided into three parts: the first part (6 items) investigated the relevant variables of learners, namely gender, experience, year of study, and dependent variable BI; the second part (23 items) investigated seven independent variables in the UTAUT2 model (Venkatesh et al., 2012; Zeng, 2019); and the third part investigated learners’ SE (8 items) and MS (12 items), with questions adapted from the motivated strategies for learning questionnaire developed by Pintrich et al. (1993). All items except those related to the relevant variables (gender, experience, and year of study) were measured using a 5-point Likert scale. All statistical analyses were conducted using SPSS v21.0 and SPSS AMOS for Windows v21.0 (IBM, Armonk, NY, United States).

## Data Analysis and Results

### Participant Information

Because of the widespread suspension of in-person classes in the universities in East China, online teaching has been extensively promoted. Therefore, questionnaires were issued to 45 universities in East China from September to December 2020 through various channels. The inclusion criteria were undergraduate students (freshmen, sophomores, juniors, or seniors) aged 19–22 years; 566 valid questionnaires were retrieved (response rate: 89.8%).

Of them, 227 (40.1%) were from male learners and 339 (59.9%) from female learners. In terms of year of study, the distribution was comprehensive, with 173 freshmen (30.6%), 168 sophomores (29.7%), 179 juniors (31.6%), and 46 seniors (8.1%) responding to the questionnaire. The majors were widely distributed, including philosophy, economics, law, education, literature, science, engineering, agriculture, medicine, management, art, and historiography. Overall, the research sample was evenly distributed in terms of gender, year of study, and major; the sample can thus represent average university learners in East China.

### Difference and Correlation

#### Analysis of Relevant Variables for Gender, Year of Study, and Experience

The Cronbach’s alpha of variables in the questionnaire were shown as Table 2, which were all above 0.7, implying high confidence. The Kaiser–Meier–Olkin measure of sampling adequacy was 0.935, which is greater than 0.70, and the results of the Bartlett’s test of sphericity was *p* < 0.01, indicating the favorable validity (Clark and Watson, 2019).

**TABLE 2:** Reliability statistics.

Variables	Cronbach’s alpha
PE	0.877
EE	0.899
SI	0.896
FC	0.918
HM	0.851
PV	0.896
Habits	0.853
BI	0.864
SE	0.929
MS	0.900

Differences in BI of different genders and learning experience were tested with an independent samples *t*-test. Significant difference were observed in BI to continue online learning among male and female learners (*p* = 0.000 < 0.01), and whether learners had experience with online learning resulted in significant differences in BI (*p* = 0.002 < 0.01; Table 3). ANOVA was used to examine the difference between the 4 years of study; no significant difference in BI was observed between the different years of study (*p* > 0.05).

**TABLE 3:** Independent samples *t*-test on BI for gender and experience.

	*t*	df	Sig. (two-tailed)	Mean difference	Std. error difference	95% Confidence interval of the difference	
						Lower	Upper
Gender	3.694	564	0.000	0.269	0.073	0.126	0.412
Experience	3.254	113.545	0.002	0.360	0.111	0.141	0.579

#### Correlations Between Variables

Correlation refers to an association between two elements that cannot be explained directly (Liang et al., 2016). The results of the correlations between the variables in the UTAUT2-based e-learning model are presented in Table 4.

**TABLE 4:** Correlations between variables.

	PE	EE	SI	FC	HM	PV	Habits	SE	MS	BI
PE	1									
EE	0.287**	1								
SI	0.447**	0.366**	1							
FC	0.433**	0.302**	0.565**	1						
HM	0.296**	0.306**	0.383**	0.321**	1					
PV	0.331**	0.308**	0.412**	0.344**	0.511**	1				
Habits	0.202**	0.219**	0.214**	0.246**	0.496**	0.504**	1			
SE	0.474**	0.396**	0.605**	0.564**	0.494**	0.544**	0.370**	1		
MS	0.192**	0.258**	0.315**	0.301**	0.295**	0.238**	0.251**	0.256**	1	
BI	0.361**	0.380**	0.480**	0.428**	0.510**	0.533**	0.473**	0.560**	0.335**	1

**Correlation is significant at the 0.01 level (two-tailed).

Correlation analysis revealed that PE, EE, SI, FC, HM, PV, habits, SE, and MS all had significant positive correlations with BI. Therefore, a multivariate analysis of these variables was performed using an SEM.

### Model Inspection and Modification

**FIGURE 3:**
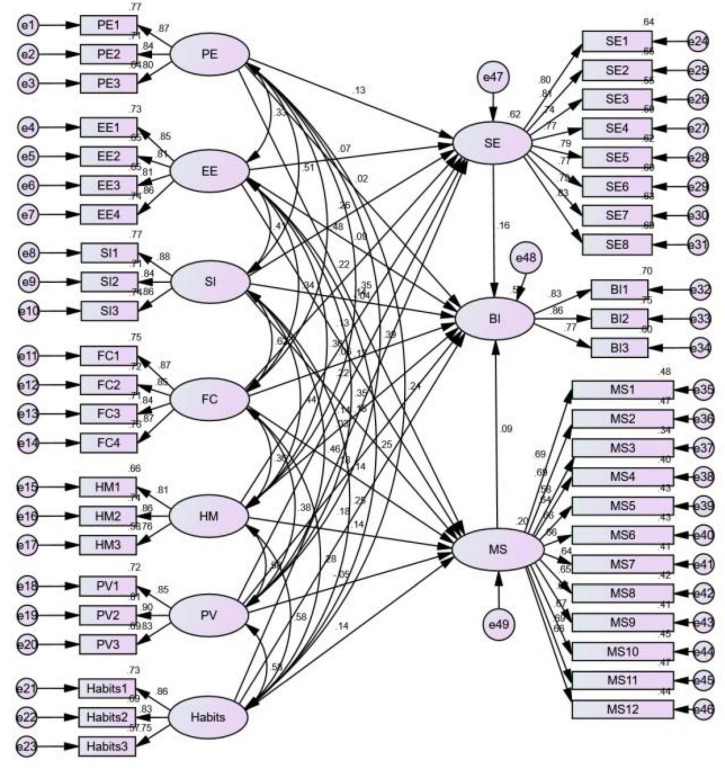
SEM of UTAUT2-based e-learning.

Structural equation modeling is a key multivariate analysis tool; in structural equation modeling, the covariance matrix of variables is used to analyze correlation (Cheng, 2006). This study applied the maximum likelihood method to implement estimates of the relevant parameters of the UTATU2-based e-learning model (Figure 3).

#### Model Fit

Fitness indices are typically used to evaluate the appropriateness of an SEM. The model exhibits a favorable fit when 2/*df* is <3, root mean square error of approximation is <0.08, and fitness indices such as the goodness of fit index, adjusted goodness of fit index, normed fit index, incremental fit index, and comparative fit index are >0.9 (values of 0.8–0.9 are considered acceptable). According to the results in Table 5, this model exhibited a favorable fit.

**TABLE 5:** Model fit.

Model	CMIN/df	GFI	AGFI	NFIDelta1	IFIDelta2	TLIRho2	CFI	RMSEA
Default model	1.702	0.887	0.872	0.895	0.954	0.949	0.953	0.035
Saturated model		1.000		1.000	1.000		1.000	
Independence model	14.894	0.185	0.151	0.000	0.000	0.000	0.000	0.157

#### Inspection of SEM

Under the influence of the relevant variables (gender, experience, and year of study), EE, SI, HM, PV, and habits had significant positive effects on BI, whereas PE and FC had no significant effect on BI (Table 6).

**TABLE 6:** Inspection of SEM.

Hypothetical relationship			Unstandardized		Standardized	Label
			Estimate	*p*	Estimate	
SE	→	BI	0.106	0.026	0.132	True
MS	→	BI	0.102	0.014	0.096	True
**PE**	→	BI	**0.029**	**0.489**	**0.031**	**False**
EE	→	BI	0.070	0.019	0.094	True
SI	→	BI	0.098	0.016	0.135	True
**FC**	→	BI	**0.039**	**0.246**	0.057	**False**
HM	→	BI	0.140	0.009	0.141	True
PV	→	BI	0.168	***	0.188	True
Habits	→	BI	0.188	***	0.184	True

Bold terms and values refer that p > 0.05 and there is no significant difference. ***p < 0.001.

#### Mediating Effect

In addition to the effect of the seven variables in the UTAUT2 model on BI, SE and MS also have significant positive effects on BI. To assess the mediating effect of these two variables on BI, this study used the bootstrap method; Table 7 presents the 14 paths.

**TABLE 7:** Mediating effects of study variables.

Parameter (standardized)	Estimate	Lower	Upper	*p*
PE–SE–BI	0.017	0.001	0.048	0.038
**EE–SE–BI**	**0.010**	**0.000**	**0.032**	**0.054**
SI–SE–BI	0.035	0.001	0.084	0.044
FC–SE–BI	0.029	0.002	0.068	0.037
HM–SE–BI	0.017	0.002	0.049	0.032
PV–SE–BI	0.029	0.002	0.072	0.042
**Habits–SE–BI**	**0.004**	**−0.007**	**0.027**	**0.350**
**PE–MS–BI**	**−0.003**	**−0.019**	**0.006**	**0.390**
EE–MS–BI	0.011	0.002	0.031	0.012
SI–MS–BI	0.015	0.002	0.040	0.012
FC–MS–BI	0.014	0.002	0.034	0.011
HM–MS–BI	0.013	0.001	0.037	0.021
**PV–MS–BI**	**−0.004**	**−0.024**	**0.008**	**0.383**
Habits–MS–BI	0.013	0.002	0.039	0.020

*Bold terms and values refer that p > 0.05 and there is no significant difference.*

Regarding SE’s mediating effect, except for the EE–SE–BI and habits–SE–BI paths (*p* > 0.05), the interval range in other mediating paths did not contain 0 (*p* < 0.05), thus indicating the existence of a mediating path. In addition, the estimate value of 0.035 indicates that SE had the largest mediating effect on the association between SI and BI.

Regarding MS’s mediating effect, except for the PE–MS–BI and PV–MS–BI paths (*p* > 0.05), the interval range in other mediating paths did not contain 0 (*p* < 0.05), thus indicating the existence of a mediating path. In addition, the estimate value of 0.015 indicates that MS had the largest mediating effect on the association between SI and BI.

#### Model Modification

Some paths in the null hypothesis model were false. EE, SI, HM, PV, and habits in the UTAUT2-based e-learning model had significant positive effects on BI, verifying the paths of EE → BI, SI → BI, HM → BI, PV → BI, and habits → BI in the null hypothesis. PE and FC had no significant effect on BI, rejecting the paths of PE → BI and CF → BI. SE had a mediating effect in the association of PE, SI, FC, H M, and PV with BI, rejecting the paths of EE → SE → BI and habits → SE → BI in the null hypothesis. MS had a mediating effect in the association of EE, SI, FC, HM, and habits with BI, rejecting the paths of PE → MS → BI and PV → MS → BI in the null hypothesis.

**FIGURE 4:**
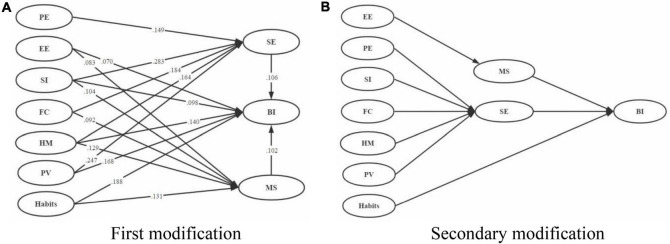
Modification of the UTAUT2-based e-learning model. **(A)** First modification. **(B)** Secondary modification.

Thus, first modifications were applied to the null hypothesis model (i.e., the UTAUT2-based e-learning model; Figure 2), and the path coefficient of each existing path is marked on the relevant lines in Figure 4A. The effects of the independent variables on the dependent variable had up to three branches. For example, the path coefficients of SI directly affecting BI was 0.098, of SI affecting BI through SE was 0.283, and of SI affecting BI through MS was 0.104. In comparison, SI had the greatest effect on BI through the mediation of SE. Similarly, EE had the greatest effect on BI through the mediation of MS, FC through the mediation of SE, HM through the mediation of SE, and PV through the mediation of SE; habits had the greatest effect on BI directly. The secondary modification to the model is presented in Figure 4B

## Discussion and Suggestions

### Attach Great Importance to the Educational Status of Online Learning and the Independent Initiative of Learners

The study results revealed that PE and FC in the UTAUT2 model had no effect on the learners’ willingness to continue online learning (BI), but the mediating effect of SE enhanced the significant effects of PE and FC on BI. The effectiveness of online learning should not be judged merely according to improvements in learning performance. The performance output of higher education is long-term work; colleges and universities should reexamine the function of online learning and focus on cultivating students’ core qualities related to their personal and social development. Packmohr and Brink (2021) found that students during the pandemic were more critical of the effects on their learning success, and this could cause great pressure on learners making low PE. Thus PE had no significant effect on learners’ BI.

The result that FC in the UTAUT2 model had no effect on the learners’ willingness to continue online learning is consistent with Tandon et al. (2021), they found that technology risk as an inhibitor emerged insignificant. However, Al-Karaki et al. (2021) believed that technical needs were related to students’ satisfaction and success in distance learning, absence of the proper infrastructure would hinder the operation especially in courses with hands-on components. This is related to the applicability of the model, which was originally applied in the commercial field, mainly to predict users’ acceptance of new technologies (Venkatesh et al., 2003). Online learning is not a new concept, although various new technologies such as artificial intelligence and mixed reality are being studied by researchers and introduced in the educational field as online learning technology continues to evolve, the technical equipment that supports students in online learning is actually exceedingly simple from the perspective of knowledge dissemination. Thus FC had no significant effect on learners’ BI. Online learning supports the bring your own device concept and diversified forms of knowledge dissemination and enables functions such as asynchronous distribution of resources and assignments; thus, online teaching can be realized in colleges and universities. For learners, online learning is easy to master without instructional materials, particularly for younger generation of learners who are digital natives.

Online learning cannot replace in-person teaching in colleges and universities, while during the COVID-19 it is a very good alternative solution to face-to-face approach (Al-Karaki et al., 2021). Improving academic performance is not the original intention of online learning, and advanced and expensive technical equipment cannot maintain learning enthusiasm over extended periods (Chen, 2021). The core of online learning is the careful design of teaching content, which front-line teachers are encouraged to explore scientifically and logically and to conduct on-demand teaching on the basis of learners’ existing knowledge, enabling learners to leverage their strengths in online learning. In other words, when learners’ SE is enhanced, PE and FC have a substantial effect on BI.

### Lower Barriers to Learn Online and Stimulate Learners’ Learning Motivation

As expected, EE, SI, HM, PV, and habits had a direct effect on BI, which were contrary to the results of Tandon et al. (2021), which showed that SI and PV did not emerge significant. Moreover, the significant effect of EE on BI was enhanced through the mediating effect of MS, and the effect of SI, HM, and PV on BI was enhanced through the mediation of SE. However, the effect of habits on BI remained very strong without the mediation of SE and MS.

During the pandemic period, some educational institutions encouraged teachers to use popular teaching tools, including some commonly used social software (e.g., QQ and WeChat) to ensure learning could proceed. In addition, network communication companies have been cooperating with universities to reduce the costs associated with online learning, and each online learning platform also provides full technical support to ensure successful learning progress. Therefore, learners can engage in online learning without changing their habits, investing additional effort (EE), or incurring high costs (PV); this is vital because lower barriers would increase learners’ BI to continue online learning. Before the pandemic, massive open online courses (MOOCs) were already changing people’s traditional view of online teaching (SI), for online learning becoming students’ preference that made the amalgamation of knowledge, human force (teacher) and technological force (Ranjan et al., 2021). Online learning is no longer the traditional form of non-formal education but a teaching form in which high-quality teaching resources are shared. Learners regard online learning as more enjoyable (HM) than face-to-face learning, thereby stimulating their learning motivation, which directly affects their BI.

Meanwhile, this study considered the added factors’ mediating effect, that was the effect of EE on BI was higher under the mediating effect of MS than when EE affected BI directly. This result indicates that online learning through familiar technology enables learners to expend less effort and focus more on how to regulate themselves to adapt to the online learning process. The enhanced learning experience does not increase their cognitive load, thus promoting their willingness to continue online learning. Similarly, the effects of SI, HM, and PV on BI were enhanced through the mediating effect of SE. This result indicates that when the value of online learning is recognized by society, learners spend less on experiencing a pleasant online learning process and believe that they have adequate social support, learning energy, and financial means to complete online learning. Thus, learners’ SE is improved, learning motivation is stimulated, and they are more willing to continue online learning in the future.

### Stimulate Positive Public Opinion and Improve the Social Recognition of Online Learning

The results revealed that compared with the other independent variables, the mediating value of SE and MS on the effect of SI on BI was the largest (0.035 and 0.015, respectively). SI refers to the view of social groups around individuals on the learners’ online learning. The external approach of learning drive involves effectively changing the learners’ motivation from “teachers want you to learn” to “you yourself want to learn” (Wang et al., 2020).

The teaching content in college and university courses is closely linked to students’ future employment. Therefore, higher education can be regarded as an economic behavior. To improve the quality of curriculum implementation, some studies have highlighted the necessity of enhancing the social and cultural environment of campuses (Chen and Miao, 2020). For positive public opinion regarding online learning, the public’s beliefs, attitudes, and emotions regarding online learning should be improved (Xu and Zhang, 2008). General social recognition directly affects the training quality and employment rate of part-time postgraduates (Li X. et al., 2020).

Similarly, a positive attitude of the general public toward online learning can effectively improve the learners’ BI. If society in general and elders in the family in particular recognize online learning as a formal type of learning, learners are more likely to realize their learning potential; this can be achieved by considering their capability of completing the complex tasks of online learning, improving their SE, or making an effort to regulate cognitive strategies to adapt to the progress of online learning to gain the praise of their elders (Cook and Ausubel, 1970). Therefore, compared with other independent variables, SI results in a greater increase in confidence related to online learning and expectation of future use of online learning through the mediating effects of SE and MS.

## Conclusion

This study used a literature review and questionnaire survey and modified the UTAUT2 model to form the UTAUT2 e-learning model to integrate the two mediating variables, SE and MS. By investigating the first round of online learning for undergraduates during the COVID-19 pandemic, the direct and mediating factors affecting learners’ willingness to continue online learning (BI) were analyzed through data analysis and structural equation modeling; on the basis of the results, the UTAUT2-based e-learning model was modified.

The results indicated that SE enhanced the effects of EE, SI, FC, HM, and PV on learners’ BI; that MS enhanced the effects of EE on learners’ BI; and that habits have a direct and strong effect on BI. This study also provided some suggestions to enhance higher education learners’ BI to continue online learning, which can serve as reference for colleges and universities to conduct, continue, or enhance online education, particularly as the pandemic continues.

This study had certain limitations. The UTAUT2 e-learning model was developed from the UTATU2 model, the discussion could only compared with the studies regarded to UTATU2 model. Meanwhile, the real thoughts of learners might be different with what examined in the questionnaire, thus more participants are needed to test the validity and accuracy of the UTAUT2 e-learning model. Future research can add different variables which may effect the learning willingness and behavior as mediating factors or dependent variables to innovate the model.

## Data Availability Statement

The raw data supporting the conclusions of this article will be made available by the authors, without undue reservation.

## Author Contributions

WX and Z-YS designed and carried out the research and conducted the data analysis and summary. S-JL and J-CC issued the questionnaires, conducted the research, and participated in the data analysis. All authors contributed to the article and approved the submitted version.

## Conflict of Interest

The authors declare that the research was conducted in the absence of any commercial or financial relationships that could be construed as a potential conflict of interest.

## Publisher’s Note

All claims expressed in this article are solely those of the authors and do not necessarily represent those of their affiliated organizations, or those of the publisher, the editors and the reviewers. Any product that may be evaluated in this article, or claim that may be made by its manufacturer, is not guaranteed or endorsed by the publisher.
